# Association of Preoperative Inspiratory Muscle Weakness and Respiratory Sarcopenia with Postoperative Pneumonia Following Esophagectomy: A Multicenter Retrospective Cohort Study

**DOI:** 10.1245/s10434-026-19625-x

**Published:** 2026-04-15

**Authors:** Kazuki Okura, Tomohiro Ikeda, Hiroki Sato, Sho Katayama, Yusuke Takahashi, Yushi Nagaki, Akiyuki Wakita, Naoaki Maeda, Shunsuke Tanabe, Yoshinori Fujiwara, Kazuhiro Noma, Yusuke Sato, Yuji Kasukawa, Naohisa Miyakoshi

**Affiliations:** 1 Department of Rehabilitation Medicine, Akita University Hospital, Akita City, Japan; 2 Department of Rehabilitation Medicine, Okayama University Hospital, Okayama City, Japan; 3 Department of Rehabilitation, Kawasaki University of Medical Welfare, Kurashiki City, Japan; 4 Department of Esophageal Surgery, Akita University Hospital, Akita City, Japan; 5 Department of Thoracic Surgery, Akita University Graduate School of Medicine, Akita City, Japan; 6 Department of Gastroenterological Surgery, Graduate School of Medicine, Dentistry and Pharmaceutical Sciences, Okayama University, Okayama City, Japan; 7 Department of Digestive Surgery, Kawasaki Medical School, Kurashiki City, Japan

**Keywords:** Inspiratory muscle, Respiratory sarcopenia, Postoperative pneumonia, Perioperative rehabilitation, Esophagectomy, Esophageal cancer

## Abstract

**Background:**

Esophagectomy is associated with a high rate of postoperative pneumonia, which significantly impacts patient outcomes, including survival and quality of life. While some modifiable risk factors have been identified, the specific role of preoperative respiratory muscle function remains to be fully elucidated. Therefore, this study was designed to investigate the association of preoperative inspiratory muscle weakness (IMW) and respiratory sarcopenia (RS) with postoperative pneumonia in patients with esophageal cancer who underwent esophagectomy.

**Methods:**

Patients with esophageal cancer who underwent esophagectomy between July 2021 and June 2023 were enrolled in this multicenter, retrospective, cohort study. The primary outcome was postoperative pneumonia, while preoperative IMW and RS were the main exposures. Respiratory sarcopenia was defined as the presence of both IMW and low skeletal muscle mass, which is assessed by using bioelectrical impedance analysis. Associations were analyzed by using G-computation within a Bayesian framework.

**Results:**

A total of 213 patients were enrolled in this study. Postoperative pneumonia occurred in 42 patients (19.7%). Preoperative IMW was strongly associated with an increased risk of pneumonia, with a mean risk difference (RD) of 18.1% (95% credible interval [CrI] 5–33.6). The posterior probability that the RD exceeds 5% was > 98%. Respiratory sarcopenia also showed a potential association, although with greater uncertainty (mean RD, 11.2%; 95% CrI − 3.8 to 27.9). The posterior probability that the RD exceeds 5% was 76.7%.

**Conclusions:**

Preoperative IMW is a notable risk factor for postoperative pneumonia following esophagectomy. While a potential link with RS was found, its role remains uncertain and requires further investigation.

**Supplementary Information:**

The online version contains supplementary material available at 10.1245/s10434-026-19625-x.

Esophagectomy is a definitive treatment for resectable esophageal cancer. Despite significant advances in surgery and supportive care, post-esophagectomy complications remain common.^1^ Among these, postoperative pulmonary complications, including pneumonia, are the most frequent and have a major clinical impact, because they increase the length of hospital stays and medical costs, and adversely affect in-hospital mortality, long-term survival, and health-related quality of life.^2–6^ Therefore, developing treatments and supportive care to reduce postoperative pneumonia is an urgent priority for patients with esophageal cancer.

Nutritional status, oral and dental conditions, physical function, exercise tolerance, physical activity, and sarcopenia are the modifiable risk factors for postoperative pneumonia following esophagectomy.^7–12^ Preoperative respiratory muscle function is also considered a modifiable risk factor associated with postoperative pneumonia in patients with esophageal cancer.^13,14^ However, the evidence for these findings remains limited. Recently, the concept of respiratory sarcopenia (RS), defined as a condition with both reduced respiratory muscle strength and low muscle mass, has been proposed.^15^ Respiratory sarcopenia has been reportedly associated with complications, including pulmonary complications, and long-term survival after lung resection^16^; however, its association with postoperative pneumonia following esophagectomy has not yet been investigated.

Therefore, this study was designed to investigate the association of preoperative inspiratory muscle weakness (IMW) and RS with postoperative pneumonia in patients with esophageal cancer who underwent esophagectomy. We specifically focused on postoperative early-phase pneumonia, which has been reported to strongly correlate with prognosis following esophagectomy.^17^ Elucidating these associations will strengthen the role of IMW as a risk factor for postoperative pneumonia and establish RS as a new modifiable risk factor in patients with esophageal cancer.

## Methods

### Study Design and Setting

This multicenter, retrospective, cohort study was conducted at three academic hospitals in Japan. Data of patients with esophageal or esophago-gastric junction cancer who underwent scheduled esophagectomy between July 2021 and June 2023 were retrospectively collected from electronic medical records.

### Ethics Approval and Consent to Participate

This study was approved by the institutional review board of Akita University Graduate School of Medicine (approval number, 3060); the Ethics Committee of the Okayama University Graduate School of Medicine, Dentistry, and Pharmaceutical Sciences and Okayama University Hospital (approval number, 2402-022); and the Ethics Committee of Kawasaki Medical School and Affiliated Hospital (approval number, 6611-00). Its protocol was conducted in accordance with the Declaration of Helsinki. Informed consent was waived owing to the study’s retrospective design. Bulletin boards in each hospital were used as an opt-out method to allow patients to decline participation in this study.

### Participants

Adult patients with esophageal or esophago-gastric junction cancer who underwent scheduled esophagectomy at three academic hospitals in Japan between July 2021 and June 2023 were included. Patients were excluded if they met any of the following criteria: age < 18 years, salvage esophagectomy, transhiatal esophagectomy, laryngopharyngeal esophagectomy, two-stage surgery, scheduled to undergo tracheostomy, sequelae (e.g., hemiplegia and aphasia) due to cerebrovascular disease, neuromuscular disease diagnosis, dementia diagnosis, or requiring assistance with activities of daily living.

### Measurements and Data Collection

#### Inspiratory Muscle Weakness and Respiratory Sarcopenia

Inspiratory muscle weakness was defined as a maximal inspiratory pressure (MIP) of <80% of the predicted value.^13^ Maximal inspiratory pressure was measured as inspiratory muscle strength within 3 days preoperatively, using a respiratory dynamometer (Autospiro AS-507, Minato Medical Science Co., Ltd., Osaka, Japan) according to the methods recommended by the European Respiratory Society.^18^ Values were also expressed as percentages of predicted values, which were calculated by using the Japanese equation.^19^

Respiratory sarcopenia was defined as the presence of both IMW and low skeletal muscle mass (LSM). This corresponds to the criteria for “probable respiratory sarcopenia” as proposed in a previous diagnostic algorithm.^15^ Skeletal muscle mass was assessed by using the appendicular skeletal muscle index (ASMI), measured by bioelectrical impedance analysis (BIA) with an InBody S10 device (InBody Japan Co., Ltd., Tokyo, Japan). Low skeletal muscle mass was defined based on the Asian Working Group for Sarcopenia (AWGS) 2019 consensus criteria, with cutoff values of < 7.0 and < 5.7 kg/m^2^ for men and women, respectively.^20^

#### Outcomes

Postoperative pneumonia that occurred within 7 days postoperatively was the primary outcome. The follow-up period for pneumonia onset was specifically limited to this timeframe owing to its strong association with prognosis^17^ and to exclude cases of pneumonia clearly caused by food aspiration.^21^ Oral intake was typically initiated at all participating facilities ≥7 days postoperatively. The onset date of pneumonia was defined as the date on which a physician initiated pharmacological treatment (the Clavien–Dindo grade ≥2).^22^ Pneumonia was defined as the presence of new or progressive infiltrates on chest radiography or computed tomography and the presence of at least one of the following two clinical features as previously reported: temperature ≥ 38 or ≤ 36 °C and white blood cell count ≤ 4000 or ≥ 10,000/μL.^13,14^ Other patients who closely met the above criteria and were clinically diagnosed with pneumonia by a physician were also included.

#### Collection of Clinical and Confounding Data

Demographic (sex, age, height, weight, body mass index, smoking status, performance status, comorbidities, blood test results, nutritional status, and pulmonary function) and tumor-specific (cancer histology, tumor location, clinical staging according to the Union for International Cancer Control eighth edition tumor–node–metastasis classification,^23^ and neoadjuvant therapy) data were collated from the patient medical records as preoperative characteristics. Smoking and comorbidity statuses were assessed by using the Brinkman index and Charlson Comorbidity Index, respectively. Data on comorbidities, including hypertension, dyslipidemia, diabetes mellitus, and chronic respiratory disease (chronic obstructive pulmonary disease, asthma, or interstitial lung disease), were also collected. Hemoglobin and serum albumin levels were obtained from the hematological data. Malnutrition was assessed by using the Global Leadership Initiative on Malnutrition (GLIM) criteria. Data on pulmonary function, including forced vital capacity (FVC), forced expiratory volume in 1 s (FEV_1_), and forced expiratory volume % in 1 s (FEV_1_/FVC), were obtained. The predicted value percentage was calculated for FVC and FEV_1_.

Operative details (surgical procedure, lymph node dissection area, reconstruction route, operative time, and blood loss volume), postoperative complications, and postoperative length of hospital stay were also collected. Postoperative pulmonary complications, including atelectasis/sputum expectoration difficulty, pneumonia, reintubation for respiratory failure, or acute respiratory distress syndrome; anastomotic leakage; recurrent laryngeal nerve palsy; arrhythmia; and surgical site infection were designated as major postoperative complications of esophagectomy and designated as the Clavien–Dindo grade ≥ 2. The exception was recurrent laryngeal nerve palsy, which was recorded as grade ≥ 1.

### Statistical Analysis

Descriptive data for numerical variables were summarized as means with standard deviations or medians with interquartile ranges and as numbers and percentages for categorical variables. The association of preoperative IMW and RS with postoperative pneumonia was investigated by using G-computation within a Bayesian generalized linear mixed model to estimate the marginal exposure effect. Markov chain Monte Carlo (MCMC) sampling was used to obtain the posterior distributions of the estimated models. Weakly informative priors were used for all parameters to minimize the influence of a prior assumption and ensure that the posterior distributions were driven by the observed data. We used a normal prior distribution with a mean of 0 and a standard deviation of 5 for the coefficients of the logistic regression model.

Posterior distributions were summarized using mean values as point estimates and 95% credible intervals (95% CrI) based on the highest posterior density region. We also extracted the probabilities of different effects from the posterior distributions. MCMC estimation in each model was performed with 5,000 iterations, using the initial 2,500 iterations as a burn-in and four chains with random initial chain values. Convergence was visually confirmed using trace plots and the Gelman–Rubin convergence diagnostic (Rhat) < 1.1 for each variable.

In the generalized linear mixed model, the fixed effect variables were IMW, LSM, and their interactions, while the random effect variable consisted of a random intercept for each institution. The following variables were included as covariates: sex, age, smoking status (Brinkman index), comorbidity status (Charlson Comorbidity Index), malnutrition (GLIM criteria), advanced cancer stage (clinical stage ≥ II), pulmonary function (FEV_1_/FVC), operative time, and recurrent laryngeal nerve palsy.

G-computation was performed by estimating the predicted distributions under different exposure scenarios, derived from the posterior distribution of the estimated model. The exposure effects of IMW (exposure to IMW alone) and RS (exposure to both IMW and LSM) were estimated relative to a scenario in which neither IMW nor LSM was present as a reference. Additionally, the mean of the risk difference (RD), relative risk (RR), and its 95% CrI was calculated from the predicted risk for each MCMC sample. We assumed a region of practical equivalence for the RD of ± 5% and calculated the posterior probability that the exposure-related RD exceeded 5% (clinically important exposure). Imputation was performed by using the random forest method if variables were missing in > 5% of all participants.^24^

As a sensitivity analysis, we investigated the exposure effect using models with fixed effects of either IMW or RS alone in the generalized linear mixed model. The random effects and covariates remained consistent with those used in the primary analysis. All statistical analyses were performed using R version 4.2.1 (R Foundation for Statistical Computing, Vienna, Austria) with the *Tidyverse* package, *Stan* via the *brms* package, *missForest,* and *bayestestR* packages.

## Results

### Participant Selection

**Fig. 1:**
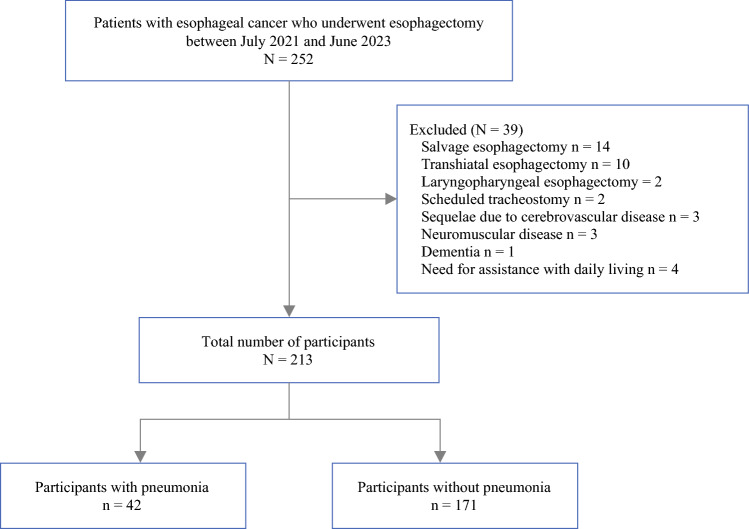
Flow diagram of participant enrollment

A total of 252 patients underwent esophagectomy during the study period. Among these, 39 were excluded based on the predefined exclusion criteria, as detailed in the flow diagram (Fig. 1). Ultimately, 213 patients were included in the final analysis.

### Exposures and Outcomes

Postoperative pneumonia, which is the primary outcome, occurred in 42 patients (19.7%) (Fig. 1). Fifteen (7%) and 24 (11.3%) patients had missing data for ASMI and MIP, respectively. Therefore, the analysis was performed after imputing these missing values. Table 1 shows the characteristics of the participants after imputation.

**Table 1:** Preoperative characteristics of the participants

Variables		Overall	RS	IMW	LSM	Other
		*N* = 213	*n* = 48	*n* = 41	*n* = 43	*n* = 81
Sex (male)	n	172 (81%)	36 (75%)	30 (73%)	36 (84%)	70 (86%)
Age	years	68 (10)	70 (9)	66 (10)	70 (10)	66 (9)
BMI	kg/m^2^	21.8 (3.2)	19.5 (2.3)	23.2 (2.9)	20.0 (2.4)	23.4 (2.9)
History of smoking	n	173 (81%)	39 (81%)	31 (76%)	36 (84%)	67 (83%)
Brinkman Index		500 (120, 840)	690 (110, 980)	700 (40, 920)	560 (200, 810)	400 (80, 780)
Comorbidity						
Hypertension	n	92 (43%)	23 (48%)	20 (49%)	22 (51%)	27 (33%)
Dyslipidemia	n	32 (15%)	10 (21%)	7 (17%)	5 (12%)	10 (12%)
Diabetes mellites	n	40 (19%)	12 (25%)	11 (27%)	6 (14%)	11 (14%)
Chronic respiratory disease	n	8 (4%)	1 (2%)	1 (2%)	4 (9%)	2 (2%)
Charlson comorbidity index		1 (0, 2)	1 (0, 2)	1 (0, 2)	0 (0, 1)	0 (0, 1)
Age-adjusted score		4 (3, 5)	5 (4, 6)	4 (3, 5)	4 (3, 5)	3 (3, 5)
ECOG performance status						
0	n	163 (77%)	24 (50%)	32 (78%)	33 (77%)	74 (91%)
≥1	n	40 (23%)	24 (50%)	9 (22%)	10 (23%)	7 (9%)
Cancer histology						
Squamous cell carcinoma	n	189 (89%)	42 (88%)	31 (76%)	40 (93%)	76 (94%)
Adenocarcinoma	n	18 (8%)	4 (8%)	7 (17%)	2 (5%)	5 (6%)
Other	n	6 (3%)	2 (4%)	3 (7%)	1 (2%)	0 (0%)
Tumor location						
Cervical esophagus	n	8 (4%)	0 (0%)	2 (5%)	3 (7%)	3 (4%)
Upper thoracic	n	32 (15%)	6 (13%)	5 (12%)	4 (9%)	17 (21%)
Middle thoracic	n	98 (46%)	32 (67%)	16 (39%)	17 (40%)	33 (41%)
Lower thoracic	n	50 (23%)	4 (8%)	11 (27%)	13 (30%)	22 (27%)
Esophagogastric junction	n	25 (12%)	6 (13%)	7 (17%)	6 (14%)	6 (7%)
Clinical stage						
I	n	62 (29%)	17 (35%)	11 (27%)	17 (40%)	17 (21%)
II	n	48 (23%)	8 (17%)	9 (22%)	9 (21%)	22 (27%)
III	n	86 (40%)	17 (35%)	19 (46%)	17 (40%)	33 (41%)
IV	n	17 (8%)	6 (13%)	2 (5%)	0 (0%)	9 (11%)
Neoadjuvant therapy	n	141 (66%)	30 (63%)	30 (73%)	21 (49%)	60 (74%)
Chemotherapy	n	119 (56%)	22 (46%)	26 (63%)	17 (40%)	54 (67%)
Chemoradiation therapy	n	22 (10%)	8 (17%)	4 (10%)	4 (9%)	6 (7%)
Hemoglobin	g/dL	11.7 (2.3)	11.7 (2.1)	11.7 (2.4)	11.3 (1.9)	11.9 (2.5)
Albumin	mg/dL	4.0 (1.6)	3.7 (1.2)	4.0 (1.5)	4.1 (1.7)	4.3 (1.8)
GNRI		98.9 (23.3)	93.0 (17.9)	103.0 (18.7)	93.3 (22.9)	103.4 (27.0)
Malnutrition (GLIM criteria)	n	59 (28%)	22 (46%)	6 (15%)	22 (51%)	9 (11%)
FVC	%predicted	102.4 (16.1)	97.0 (18.5)	101.5 (15.7)	103.2 (16.6)	105.6 (13.9)
FEV_1_	%predicted	90.3 (24.2)	89.1 (25.3)	87.8 (24.1)	86.7 (31.4)	94.2 (18.4)
FEV_1_/FVC	%	75.4 (9.7)	75.1 (10.6)	76.6 (7.8)	74.6 (13.0)	75.4 (7.8)
ASMI	kg/m^2^	6.8 (1.0)	6.0 (0.8)	7.2 (0.9)	6.2 (0.6)	7.4 (0.7)
MIP	cmH_2_O	64.7 (22.7)	42.7 (11.8)	52.2 (12.9)	67.5 (16.8)	82.5 (18.9)
	%predicted	87.7 (25.8)	63.3 (13.8)	66.7 (14.1)	102.4 (19.4)	104.9 (19.0)

Statistics: n (%), mean (standard deviation) or median (1st quartile, 3rd quartile). Percentages may not total 100 because of rounding

*RS* respiratory sarcopenia; *IMW* inspiratory muscle weakness; *LSM* low skeletal muscle mass; *BMI* body mass index; *ECOG* Eastern cooperative oncology group; C-reactive protein; *GNRI* geriatric nutrition risk index; *GLIM* Global Leadership Initiative on Malnutrition; *FVC* forced vital capacity; *FEV1* forced expiratory volume in one second; *ASMI* appendicular skeletal muscle index; *MIP* maximal inspiratory pressure

Among the complete cases, which included 184 patients, 74 (40.2%), 76 (41.4%), and 38 (20.7%) had preoperative IMW, LSM, and RS, respectively (Table S1). Postoperative pneumonia occurred in 21 (28.4%) of the patients with preoperative IMW, 11 (28.9%) of those with preoperative RS, and seven (9.9%) of those without either IMW or LSM. Among the 74 patients with IMW, 36 had IMW alone (i.e., without RS), and postoperative pneumonia occurred in ten (27.8%) of these patients (Table S2).

### Relationships Between Preoperative Inspiratory Muscle Weakness, Respiratory Sarcopenia, and Postoperative Pneumonia

The mean RD and RR for preoperative IMW were 18.1% (95% CrI 5–33.6) and 2.75 (95% CrI 1.25–4.83), respectively. Additionally, the posterior probability that the RD for preoperative IMW exceeds 5% was 98.1%.

For preoperative RS, the mean RD and RR were 11.2% (95% CrI −3.8 to 27.9) and 1.93 (95% CrI 0.67–3.63), respectively. The posterior probability that the RD for preoperative RS exceeds 5% was 76.9% (Table 2).

**Table 2:** Intra- and postoperative information of the participants

Variables		Overall	RS	IMW	LSM	Other
		*N* = 213	*n* = 48	*n* = 41	*n* = 43	*n* = 81
Surgical procedure						
Robot-assisted thoracoscopic	n	101 (47%)	23 (48%)	21 (51%)	17 (40%)	40 (49%)
Thoracoscopic	n	106 (50%)	25 (52%)	17 (41%)	25 (58%)	39 (48%)
Open thoracic	n	6 (3%)	0 (0%)	3 (7%)	1 (2%)	2 (2%)
Lymphadenectomy						
Three-field	n	126 (59%)	31 (65%)	28 (68%)	15 (35%)	52 (64%)
Two-field	n	83 (39%)	16 (33%)	13 (32%)	27 (63%)	27 (33%)
Other	n	4 (2%)	1 (2%)	0 (0%)	1 (2%)	2 (3%)
Reconstructive route						
Posterior mediastinal	n	68 (32%)	13 (27%)	12 (29%)	16 (37%)	27 (33%)
Retrosternal	n	126 (59%)	30 (63%)	26 (63%)	21 (49%)	49 (60%)
Other	n	19 (9%)	5 (10%)	3 (7%)	6 (14%)	5 (6%)
Surgery time	min	640 (557, 716)	634 (542, 715)	666 (568, 731)	623 (560, 684)	650 (559, 715)
Bleeding	mL	150 (70, 246)	123 (55, 213)	150 (70, 237)	180 (70, 450)	130 (79, 213)
Postoperative complication						
Pneumonia	n	42 (20%)	17 (35%)	12 (29%)	5 (12%)	8 (10%)
Pulmonary complications	n	59 (28%)	22 (46%)	15 (37%)	10 (23%)	12 (15%)
Anastomotic leakage	n	10 (5%)	2 (4%)	1 (2%)	1 (2%)	6 (7%)
Recurrent laryngeal nerve palsy	n	30 (14%)	8 (17%)	4 (10%)	9 (21%)	9 (11%)
Surgical site infection	n	9 (4%)	1 (2%)	4 (10%)	2 (5%)	2 (2%)
Arrhythmia	n	11 (5%)	4 (8%)	1 (2%)	1 (2%)	5 (6%)
Postoperative length of hospital stays	days	23 (19, 31)	28 (23, 39)	25 (20, 34)	23 (19, 33)	22 (18, 26)

Statistics: n (%) or median (1st quartile, 3rd quartile). Percentages may not total 100 because of rounding

*RS* respiratory sarcopenia; *IMW* inspiratory muscle weakness; *LSM* low skeletal muscle mass

**Fig. 2:**
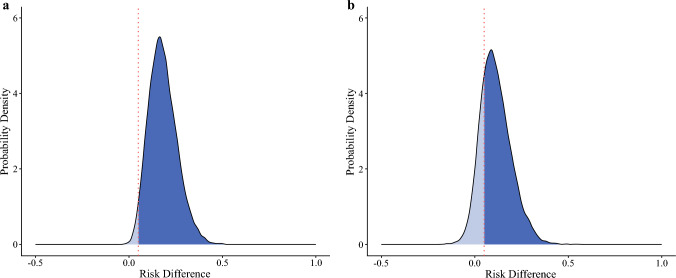
Density plot of the posterior distribution for the risk difference. The vertical red dot line indicates a risk difference of 0.05 (5%), and the probability that the risk difference >5% is the blue-filled area in the density plot. The posterior probability that the risk difference exceeds 5% for preoperative IMW (**a**) and RS (**b**) was 98.1% and 76.9%, respectively. The mean risk difference for preoperative IMW and RS was 18.1% (95% CrI 5.0–33.6) and 11.2% (95% CrI − 3.8 to 27.9), respectively. *IMW* inspiratory muscle weakness; *RS* respiratory sarcopenia; *CrI* credible interval

**Fig. 3:**
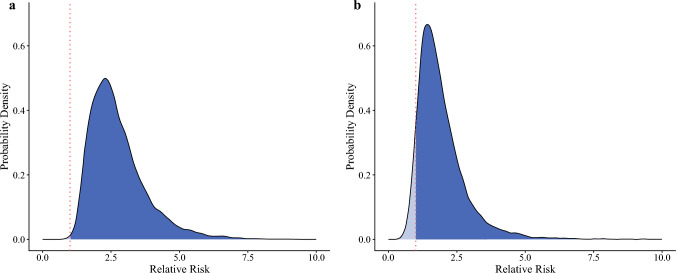
Density plot of the posterior distribution for the relative risk. The vertical red dot line indicates a relative risk of 1, and the probability that the relative risk < 1 is the blue-filled area in the density plot. The mean relative risk for preoperative IMW (**a**) and RS (**b**) was 2.75 (95% CrI 1.25–4.83) and 1.93 (95% CrI 0.67–3.63), respectively. *IMW* inspiratory muscle weakness; *RS* respiratory sarcopenia; *CrI* credible interval

Figures 2 and 3 show the density plots of the posterior distributions for the RD and RR of preoperative IMW and RS, respectively. In all models, the values of Rhat for the independent variable were equal to 1.0, indicating convergence across the four chains. The trace plots for the fixed effect variables (i.e., IMW, LSM, and their interactions) were shown in Fig. S1.

### Sensitivity Analysis

The mean RD and RR for preoperative IMW were 17.2% (95% CrI 3.9% to 31.6%) and 2.56 (95% CrI 1.17–4.34), respectively. Moreover, the posterior probability that the RD for preoperative IMW exceeds 5% was 97%.

For preoperative RS, the mean RD and RR were 8.7% (95% CrI −3.7 to 22.6) and 1.61 (95% CrI 0.77–2.61), respectively. The posterior probability that the RD for preoperative RS exceeds 5% was 67.2%.

## Discussion

This study primarily aimed to clarify the association of preoperative IMW and RS with the incidence of postoperative pneumonia following esophagectomy. Our main findings indicate a strong association between preoperative IMW and an increased risk of postoperative pneumonia. The mean RD and RR were 18.1% and 2.75, respectively, and the posterior probability of a clinically important exposure (RD > 5%) was 98.1%. Preoperative RS showed a potential association with postoperative pneumonia, although with greater uncertainty than IMW. The mean RD and RR were 11.2% and 1.93, respectively, while the posterior probability of a clinically important exposure was 76.9%.

This study’s results provide strong evidence for the association between preoperative IMW and postoperative pneumonia in patients with esophageal cancer. The mean RD of 18.1% suggests that one additional patient would experience postoperative pneumonia for every five patients with preoperative IMW. As previously reported, inspiratory muscle strength and the function of the diaphragm, which is the primary inspiratory muscle, decline following esophagectomy.^14,25,26^ Postoperatively, the inspiratory muscles must counteract the decreased respiratory compliance for various reasons. Therefore, a decline in inspiratory muscle function can lead to poor lung re-expansion and airway clearance, which may be associated with postoperative pneumonia. Given this context, preoperative inspiratory muscle training (IMT) could help to prevent pulmonary complications in patients with reduced inspiratory muscle function. A recent meta-analysis concluded that preoperative inspiratory muscle strengthening does not reduce postoperative pulmonary complications in patients with esophageal cancer.^27^ Nevertheless, the effect was in the direction of reduction (pooled odds ratio 0.57; 95% confidence interval 0.30–1.07),^27^ suggesting the need to identify more effective target populations. Our finding that IMW is a notable risk factor suggests that it serves as a key indicator for selecting such a population. Attempts have been made to implement IMT starting during the neoadjuvant therapy, indicating that longer-term preventive interventions are useful for reducing pulmonary complications.^28^ Preoperative assessment of inspiratory muscle function as a risk factor for pulmonary complications helps identify patients who would benefit from more intensive perioperative respiratory care (e.g., more frequent physiotherapy).^29–31^

Regarding the association between RS and postoperative pneumonia, two findings were obtained as follows: (1) the incidence rate of pneumonia was comparable to that in the IMW group (28.9% vs. 28.4%), and (2) its potential as a risk factor for postoperative pneumonia remains unclear compared with IMW. No standardized method has yet been established for measuring respiratory muscle mass, a key component of the RS. In this study, definition of RS correspond to "probable respiratory sarcopenia," as we used skeletal muscle mass as a surrogate for respiratory muscle mass, based on the proposed algorithm.^15^ This definition may explain why RS did not provide additional risk for postoperative pneumonia beyond IMW alone. LSM, defined by the AWGS 2019 criteria, is highly prevalent among patients with esophageal cancer but is not associated with postoperative pneumonia or survival.^32,33^ The inclusion of nutritional status as an important confounding factor—for which LSM is an indicator in the GLIM criteria—may also have influenced the observed association between RS and postoperative pneumonia.^34^ Considering the characteristics of esophageal cancer as the target disease, a more specific measurement of respiratory muscle mass is needed. For example, ultrasound-based measurement of diaphragm thickness has been suggested as a possible indicator.^15^ Previous studies have reported associations between diaphragm thickness or its thickening fraction and the occurrence of pulmonary complications following esophageal or lung cancer surgery.^14,35^ Therefore, using these measures may provide a more useful risk indicator. A single-center study reported that RS, defined by low diaphragm thickness and IMW, was associated with postoperative pulmonary complications.^36^ Future research should verify whether this combined indicator is a stronger risk factor than IMW alone and assess its external validity.

The strength of this multicenter study was the finding that IMW—a parameter easily accessible and modifiable in clinical practice—is associated with pneumonia following esophagectomy pneumonia. Respiratory sarcopenia, when defined using skeletal muscle mass as a surrogate for respiratory muscle mass, did not show superiority over IMW as a risk factor of postoperative pneumonia. Therefore, for preoperative assessment of esophageal cancer, IMW, as a more comprehensive indicator, is considered superior for the practical assessment of the risk of postoperative pneumonia.

This study has some limitations that must be acknowledged. First, it was a retrospective study. The proportion of missing data was relatively low, with the highest being just over 10%; however, its presence in the exposure variables could introduce information bias. Although we attempted to mitigate this bias by imputing missing values using a random forest method, residual bias cannot be entirely ruled out. Second, this study may be exposed to information bias associated with the use of electronic medical records. A particular concern was the potential for variation in the diagnosis of postoperative pneumonia among physicians. We established objective diagnostic criteria incorporating the Clavien–Dindo classification and data, such as laboratory test results, to minimize this discrepancy. Despite these efforts, information bias cannot be completely ruled out for other information not specifically collected for the study purpose. This represents a limitation of the study design. Finally, while we conducted this study at three academic hospitals to enhance external validity, the findings are based on a relatively small sample size. This may limit the generalizability of our results to other populations. The limited number of patients with preoperative RS may have particularly contributed to the greater uncertainty in its estimated effect on postoperative pneumonia.

## Conclusion

This study confirmed that preoperative IMW is a notable risk factor for postoperative pneumonia following esophagectomy. Although a potential association with RS was observed, its role remains uncertain and requires further investigation to establish it as a modifiable risk factor.

## Supplementary Information

Below is the link to the electronic supplementary material.

Supplementary file1 (DOCX 26 kb)

Supplementary file2 (DOCX 21 kb)

Supplementary file3 (DOCX 2802 kb)
