# Supplementary material for: Association of Preoperative Inspiratory Muscle Weakness and Respiratory Sarcopenia with Postoperative Pneumonia Following Esophagectomy: A Multicenter Retrospective Cohort Study
Source: Ann Surg Oncol. 2026 Apr 15;33(7):6296–305. doi: 10.1245/s10434-026-19625-x (PMC13242444; doi:10.1245/s10434-026-19625-x)
Supplement: Supplementary file 1 — Supplementary file1 (DOCX 26 kb) [file 10434_2026_19625_MOESM1_ESM.docx]

Table S1. Characteristics of the complete cases

|  |  | Overall | RS | IMW | LSM | Other |
| --- | --- | --- | --- | --- | --- | --- |
| Variables |  | N = 184 | n = 38 | n = 36 | n = 38 | n = 72 |
| Sex (male) | n | 151 (82%) | 29 (76%) | 26 (72%) | 31 (82%) | 65 (90%) |
| Age | years | 67 (10) | 68 (8) | 66 (10) | 70 (11) | 66 (9) |
| BMI | kg/m^2^ | 21.8 (3.2) | 19.3 (2.3) | 23.0 (2.9) | 19.7 (2.1) | 23.6 (2.8) |
| History of smoking | n | 151 (82%) | 33 (87%) | 27 (75%) | 31 (82%) | 60 (83%) |
| Brinkman Index |  | 500(175,820) | 690(180,960) | 734(110,950) | 550(200,810) | 400(120,745) |
| Comorbidity |  |  |  |  |  |  |
| Hypertension | n | 76 (41%) | 17 (45%) | 15 (42%) | 20 (53%) | 24 (33%) |
| Dyslipidemia | n | 28 (15%) | 7 (18%) | 7 (19%) | 5 (13%) | 9 (13%) |
| Diabetes mellites | n | 31 (17%) | 7 (18%) | 8 (22%) | 6 (16%) | 10 (14%) |
| Chronic respiratory disease | n | 5 (3%) | 0 (0%) | 1 (3%) | 3 (8%) | 1 (1%) |
| Charlson comorbidity index |  | 0(0,2) | 2(0,2) | 1(0,2) | 0(0,1) | 0(0,1) |
| Age-adjusted score |  | 4(3,5) | 5(3,7) | 4(3,5) | 4(3,5) | 3(3,5) |
| ECOG performance status |  |  |  |  |  |  |
| 0 | n | 145 (79%) | 22 (58%) | 29 (81%) | 29 (76%) | 65 (90%) |
| ≥1 | n | 39 (21%) | 16 (42%) | 7 (20%) | 9 (23%) | 7 (10%) |
| Cancer histology |  |  |  |  |  |  |
| Squamous cell carcinoma | n | 162 (88%) | 34 (89%) | 26 (72%) | 35 (92%) | 67 (93%) |
| Adenocarcinoma | n | 17 (9%) | 3 (8%) | 7 (19%) | 2 (5%) | 5 (7%) |
| Other | n | 5 (3%) | 1 (3%) | 3 (8%) | 1 (3%) | 0 (0%) |
| Tumor location |  |  |  |  |  |  |
| Cervical esophagus | n | 5 (3%) | 0 (0%) | 1 (3%) | 2 (5%) | 2 (3%) |
| Upper thoracic | n | 31 (17%) | 5 (13%) | 5 (14%) | 4 (11%) | 17 (24%) |
| Middle thoracic | n | 84 (46%) | 26 (68%) | 14 (39%) | 16 (42%) | 28 (39%) |
| Lower thoracic | n | 40 (22%) | 2 (5%) | 9 (25%) | 10 (26%) | 19 (26%) |
| Esophagogastric junction | n | 24 (13%) | 5 (13%) | 7 (19%) | 6 (16%) | 6 (8%) |
| Clinical stage |  |  |  |  |  |  |
| I | n | 50 (27%) | 14 (37%) | 7 (19%) | 15 (39%) | 14 (19%) |
| II | n | 46 (25%) | 8 (21%) | 9 (25%) | 8 (21%) | 21 (29%) |
| III | n | 73 (40%) | 11 (29%) | 18 (50%) | 15 (39%) | 29 (40%) |
| IV | n | 15 (8%) | 5 (13%) | 2 (6%) | 0 (0%) | 8 (11%) |
| Neoadjuvant therapy | n | 124 (67%) | 24 (63%) | 29 (81%) | 18 (47%) | 53 (74%) |
| Chemotherapy | n | 102 (55%) | 16 (42%) | 25 (69%) | 14 (37%) | 47 (65%) |
| Chemoradiation therapy | n | 22 (12%) | 8 (21%) | 4 (11%) | 4 (11%) | 6 (8%) |
| Hemoglobin | g/dL | 11.6 (2.3) | 11.7 (2.2) | 11.5 (2.4) | 11.2 (1.9) | 11.9 (2.5) |
| Albumin | mg/dL | 4.1 (1.7) | 3.8 (1.3) | 4.0 (1.6) | 4.1 (1.8) | 4.3 (1.9) |
| GNRI |  | 98.5 (24.6) | 92.8 (19.2) | 101.6 (19.5) | 92.2 (24.0) | 103.2 (28.5) |
| Malnutrition (GLIM criteria) | n | 49 (27%) | 17 (45%) | 5 (14%) | 19 (50%) | 8 (11%) |
| FVC | %predicted | 103.0 (16.4) | 96.7 (19.1) | 102.4 (16.2) | 103.6 (17.4) | 106.2 (13.6) |
| FEV_1_ | %predicted | 91.2 (23.2) | 88.4 (25.5) | 90.1 (21.1) | 86.1 (33.0) | 95.9 (14.9) |
| FEV_1_/FVC | % | 75.6 (9.7) | 75.5 (11.4) | 75.9 (7.0) | 74.8 (13.4) | 75.8 (7.5) |
| ASMI | kg/m^2^ | 6.9 (1.0) | 6.1 (0.7) | 7.2 (0.9) | 6.2 (0.6) | 7.5 (0.7) |
| MIP | cmH_2_O | 66.4 (23.5) | 42.7 (12.5) | 51.7 (13.6) | 68.8 (17.4) | 85.0 (18.3) |
|  | %predicted | 89.4 (27.2) | 62.0 (14.7) | 65.7 (14.5) | 104.9 (19.4) | 107.4 (18.6) |
| Statistics: n (%), mean (standard deviation) or median (1st quartile, 3rd quartile). Percentages may not total 100 because of rounding.  Abbreviations: RS, respiratory sarcopenia; IMW, inspiratory muscle weakness; LSM, low skeletal muscle mass; BMI, body mass index; ECOG, Eastern cooperative oncology group; C-reactive protein; GNRI, geriatric nutrition risk index; GLIM, Global Leadership Initiative on Malnutrition; FVC, forced vital capacity; FEV_1_, forced expiratory volume in one second; ASMI, appendicular skeletal muscle index; MIP, maximal inspiratory pressure | | | | | | |
