# Supplementary material for: Association of Preoperative Inspiratory Muscle Weakness and Respiratory Sarcopenia with Postoperative Pneumonia Following Esophagectomy: A Multicenter Retrospective Cohort Study
Source: Ann Surg Oncol. 2026 Apr 15;33(7):6296–305. doi: 10.1245/s10434-026-19625-x (PMC13242444; doi:10.1245/s10434-026-19625-x)
Supplement: Supplementary file 2 — Supplementary file2 (DOCX 21 kb) [file 10434_2026_19625_MOESM2_ESM.docx]

Table S2. Intra- and postoperative information of the complete cases

|  |  | Overall | RS | IMW | LSM | Other |
| --- | --- | --- | --- | --- | --- | --- |
| Variables |  | N = 184 | n = 38 | n = 36 | n = 38 | n = 72 |
| Surgical procedure |  |  |  |  |  |  |
| Robot-assisted thoracoscopic | n | 88 (48%) | 18 (47%) | 19 (53%) | 14 (37%) | 37 (51%) |
| Thoracoscopic | n | 90 (49%) | 20 (53%) | 14 (39%) | 23 (61%) | 33 (46%) |
| Open thoracic | n | 6 (3%) | 0 (0%) | 3 (8%) | 1 (3%) | 2 (3%) |
| Lymphadenectomy |  |  |  |  |  |  |
| Three-field | n | 111 (60%) | 24 (63%) | 26 (72%) | 14 (37%) | 47 (65%) |
| Two-field | n | 70 (38%) | 13 (34%) | 10 (28%) | 23 (61%) | 24 (33%) |
| Other | n | 3 (2%) | 1 (3%) | 0 (0%) | 1 (3%) | 1 (1%) |
| Reconstructive route |  |  |  |  |  |  |
| Posterior mediastinal | n | 57 (31%) | 11 (29%) | 9 (25%) | 13 (34%) | 24 (33%) |
| Retrosternal | n | 111 (60%) | 24 (63%) | 24 (67%) | 20 (53%) | 43 (60%) |
| Other | n | 16 (9%) | 3 (8%) | 3 (8%) | 5 (14%) | 5 (7%) |
| Surgery time | min | 639(556,714) | 619(542,692) | 671(560,735) | 610(560,664) | 657(560,719) |
| Bleeding | mL | 148(70,260) | 123(50,250) | 145(70,239) | 180(100,311) | 133(75,218) |
| Postoperative complication |  |  |  |  |  |  |
| Pneumonia | n | 32 (17%) | 11 (29%) | 10 (28%) | 4 (11%) | 7 (10%) |
| Pulmonary complications | n | 48 (26%) | 15 (39%) | 13 (36%) | 9 (24%) | 11 (15%) |
| Anastomotic leakage | n | 9 (5%) | 1 (3%) | 1 (3%) | 1 (3%) | 6 (8%) |
| Recurrent laryngeal nerve palsy | n | 28 (15%) | 7 (18%) | 4 (11%) | 8 (21%) | 9 (13%) |
| Surgical site infection | n | 8 (4%) | 1 (3%) | 4 (11%) | 1 (3%) | 2 (3%) |
| Arrhythmia | n | 10 (5%) | 3 (8%) | 1 (3%) | 1 (3%) | 5 (7%) |
| Postoperative length of hospital stays | days | 23(19,31) | 27(22,33) | 25(20,34) | 23(19,34) | 22(18,26) |
| Statistics: n (%) or median (1st quartile, 3rd quartile). Percentages may not total 100 because of rounding.  Abbreviations: RS, respiratory sarcopenia; IMW, inspiratory muscle weakness; LSM, low skeletal muscle mass | | | | | | |
