# Supplementary material for: Association of Preoperative Inspiratory Muscle Weakness and Respiratory Sarcopenia with Postoperative Pneumonia Following Esophagectomy: A Multicenter Retrospective Cohort Study
Source: Ann Surg Oncol. 2026 Apr 15;33(7):6296–305. doi: 10.1245/s10434-026-19625-x (PMC13242444; doi:10.1245/s10434-026-19625-x)
Supplement: Supplementary file 3 — Supplementary file3 (DOCX 2802 kb) [file 10434_2026_19625_MOESM3_ESM.docx]

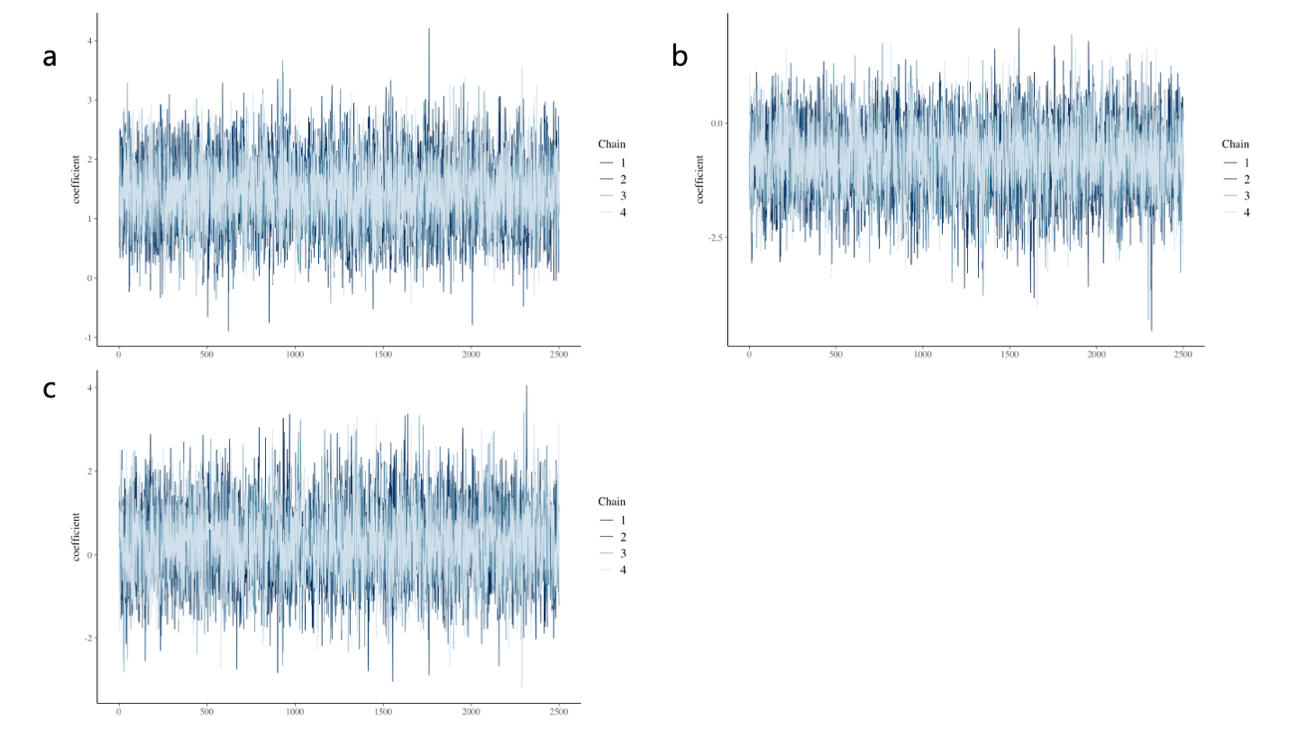


Figure S1. Trace plots after burn-in for the fixed effect variables.

a) is a trace plot in the coefficients of the inspiratory muscle weakness, b) is the coefficients of the low skeletal muscle mass, and c) is the coefficients of the interaction between inspiratory muscle weakness and low skeletal muscle mass in the generalized linear mixed effects model.
